# What can National TB Control Programmes in low- and middle-income countries do to end tuberculosis by 2030?

**DOI:** 10.12688/f1000research.14821.1

**Published:** 2018-07-05

**Authors:** Anthony D. Harries, Yan Lin, Ajay M.V. Kumar, Srinath Satyanarayana, Kudakwashe C. Takarinda, Riitta A. Dlodlo, Rony Zachariah, Piero Olliaro

**Affiliations:** 1 International Union Against Tuberculosis and Lung Disease, 68 Boulevard Saint Michel, 75006 Paris, France; 2 London School of Hygiene and Tropical Medicine, Keppel Street, London, WC1E 7HT, UK; 3 International Union Against Tuberculosis and Lung Disease, No. 1 Xindong Road, 100600 Beijing, China; 4 International Union Against Tuberculosis and Lung Disease, South-East Asia Regional Office, C-6 Qutub Institutional Area, 110016 New Delhi, India; 5 AIDS & TB Department, Ministry of Health and Child Care, 2nd Floor, Mukwati Building, Corner Livingstone Avenue and 5th Street, Harare, Zimbabwe; 6 Special Programme for Research and Training in Tropical Disease (TDR), World Health Organization, Avenue Appia 20, 1211 Geneva 27, Switzerland

**Keywords:** tuberculosis; world health organization; sustainable development goals; ending the TB epidemic; national tuberculosis programmes; diagnosis; anti-tuberculosis treatment; latent tuberculosis infection

## Abstract

The international community has committed to ending the tuberculosis (TB) epidemic by 2030. This will require multi-sectoral action with a focus on accelerating socio-economic development, developing and implementing new tools, and expanding health insurance coverage. Within this broad framework, National TB Programmes (NTPs) are accountable for delivering diagnostic, treatment, and preventive services. There are large gaps in the delivery of these services, and the aim of this article is to review the crucial activities and interventions that NTPs must implement in order to meet global targets and milestones that will end the TB epidemic. The key deliverables are the following: turn End TB targets and milestones into national measurable indicators to make it easier to track progress; optimize the prompt and accurate diagnosis of all types of TB; provide rapid, complete, and effective treatment to all those diagnosed with TB; implement and monitor effective infection control practices; diagnose and treat drug-resistant TB, associated HIV infection, and diabetes mellitus; design and implement active case finding strategies for high-risk groups and link them to the treatment of latent TB infection; engage with the private-for-profit sector; and empower the Central Unit of the NTP particularly in relation to data-driven supportive supervision, operational research, and sustained financing. The glaring gaps in the delivery of TB services must be remedied, and some of these gaps will require new paradigms and ways of working which include patient-centered and higher-quality services. There must also be fast-track ways of incorporating new diagnostic, treatment, and prevention tools into program activities so as to rapidly reduce TB incidence and mortality and meet the goal of ending TB by 2030.

## Introduction

*If you always do what you always did, you will always get what you always got.*

Albert Einstein

The Sustainable Development Goals (SDGs), adopted by United Nations (UN) Member States in September 2015, and the World Health Organization (WHO) End TB Strategy, endorsed by the WHO’s 194 Member States in 2014, have a common goal with respect to tuberculosis (TB): to end the global TB epidemic. Targets for meeting this goal include a 90% reduction in TB deaths and an 80% reduction in TB incidence by 2030 compared with 2015: these targets also need to be achieved with no TB-affected families facing catastrophic costs ^1, 2^. Milestones have been set to help monitor progress along the way ( Table 1).

**Table 1.** Global milestones and targets for ending tuberculosis by 2030.

Indicators	Milestones		Targets	
	2020	2025	SDGs 2030	End TB 2035
Percentage reduction in the absolute number of TB deaths (compared with 2015 baseline)	35%	75%	90%	95%
Percentage reduction in the TB incidence rate (compared with 2015 baseline)	20%	50%	80%	90%
Percentage of TB-affected households experiencing catastrophic costs due to TB (level in 2015 unknown)	0%	0%	0%	0%

SDG, Sustainable Development Goal; TB, tuberculosis. Adapted from reference 2.

Since the WHO took the extraordinary step in 1993 of declaring TB a “global emergency”, huge steps have been taken to control this ancient disease, once coined “the captain of all these men of death” ^3^. The “DOTS” strategy launched by the WHO in 1995, which evolved into the “Stop TB” Strategy a decade later and then the “End TB” Strategy, has provided the successive frameworks and targets for international and national control efforts. In 2000, the Millennium Development Goals (the predecessor to the SDGs) included a TB-specific target (6c) “to halt and reverse TB incidence” by 2015, and this was augmented by two Stop TB Partnership targets to halve TB prevalence and mortality by 2015 compared with levels in 1990. By 2015, TB incidence was halted and reversed, and TB-related mortality (deaths per 100,000 population per year) was decreased by 47% overall between 1990 and 2014 ^4^. TB treatment averted an estimated 49 million deaths globally between 2000 and 2015 ^4^.

Despite this laudable progress, the disease remains an enormous global public health threat, especially for low- and middle-income countries. In 2016, an estimated 10.4 million new people developed TB and 1.7 million died, making TB the ninth leading cause of death worldwide and the leading cause from a single infectious agent, ranking above HIV/AIDS ^5^. Critical problems remain at large. TB control depends on preventing latent TB infection from becoming established or developing into active disease and on diagnosing and treating active disease as soon as it has developed. TB preventive treatment is expanding, but most of those eligible for this intervention are not accessing it. In 2016, only 6.3 million new patients with TB were reported, meaning that 4.1 million (40% of the estimated disease burden) either were not diagnosed or were diagnosed but not notified to national programs ^5^. Drug-resistant TB grows inexorably. In 2016, there were an estimated 600,000 new patients with resistance to rifampicin (RR-TB), of which 490,000 had resistance to both rifampicin and isoniazid, the two most-effective first-line drugs (multidrug-resistant TB, or MDR-TB). Only 22% of patients with MDR-TB were started on treatment, and of these only 54% were successfully treated. In 2016, HIV-associated TB accounted for 1.03 million patients and was responsible for 374,000 deaths ^5^.

Ending the TB epidemic will require multi-sectoral action with a focus on accelerating socio-economic development, developing a new TB vaccine, producing novel diagnostics and medicines for treatment, and expanding health insurance coverage ^6^. The years 2017 and 2018 were and are respectively landmarks for global efforts to end TB in the era of the SDGs. In Moscow in November 2017, the WHO hosted the first global Ministerial Conference on TB and the aim was to accelerate the implementation of the WHO End TB Strategy ^5^. September 2018 will see the first UN General Assembly high-level meeting on TB at which a multi-sectoral approach and accountability framework will be agreed upon and endorsed by heads of state.

Within this broad framework, National TB Programmes (NTPs) are responsible and accountable for delivering high-quality and effective diagnostic, treatment, and preventive services and ultimately interrupting the transmission of *Mycobacterium tuberculosis* ( *MTB*) from infected to non-infected persons ^7^. To this end, the Stop TB Partnership, inspired by the Joint United Nations Programme on HIV/AIDS (UNAIDS) 90-90-90 treatment targets for HIV care and treatment ^8^, in 2015 launched 90-(90)-90 TB diagnostic and treatment targets as part of the Global Plan to accelerate the ending of the TB epidemic ( Table 2) ^9^. The aim of this article is to review the crucial activities and interventions that NTPs need to implement in order to meet the 90-(90)-90 TB targets and end the TB epidemic.

**Table 2.** 90-(90)-90 People-centered global targets for tuberculosis.

• Reach and treat at least 90% of all people with TB ^a^ • As a part of this approach, reach and treat at least (90%) of the key populations ^b^ • Achieve at least 90% treatment success for all people diagnosed with TB ^c^

TB, tuberculosis. ^a^Includes people with both drug-susceptible and drug-resistant TB as well as people who require preventive therapy (for example, people living with HIV and those in contact with patients with TB). ^b^Includes vulnerable, underserved, and at-risk populations which vary depending on country context. ^c^Includes achieving 90% treatment success among people diagnosed with both drug-susceptible and drug-resistant TB as well as people who require TB preventive therapy. Adapted from reference 9.

## Turn End TB milestones and targets into measurable numbers

NTPs must work with the WHO to develop, at the national level, absolute numbers for milestones and targets on estimated TB incidence and mortality for 2020, 2025, and 2030 so that these become easily understandable and visible indicators against which genuine progress can be measured. In the next eight years, for example, the annual decline in TB incidence has to accelerate from the current 1.5% per year to 5% per year by 2020 and to 10% per year by 2025 ^5^. The proportion of people who die from TB likewise needs to decrease to 10% by 2020 and to 6.5% by 2025. Translation of global percentage reductions to country target numbers allows NTPs to have benchmarks and to determine in detail how their states, provinces, regions, and districts are performing. Countries that are lagging behind should be given additional support to hasten progress, while those that are doing well need encouragement and praise. This approach is not new and was adopted by Malawi in 2004 when it successfully implemented the WHO’s “3 by 5” initiative for scaling up antiretroviral therapy (ART) country-wide ^10^.

## Optimize systems for prompt and accurate diagnosis of tuberculosis

There are two prongs to the diagnosis of TB: accurate identification of the disease and ascertainment of the drug-susceptibility status of *MTB.* Microbiological examination of sputum smears for acid-fast bacilli, with or without chest radiography, has long been the mainstay of TB diagnosis in resource-poor countries. However, because this method is diagnostically insensitive and cannot be used to assess drug-susceptibility status, molecular technology is rapidly taking over. The WHO-preferred test is the Xpert MTB/RIF assay (Cepheid Inc., Sunnyvale, CA, USA). This is a highly sensitive, fully-automated, and commercially available nucleic acid amplification test for use with sputum and other bodily specimens ^11^. The assay requires minimal expertise and has a short sample processing time of 2 hours to confirm the presence of *MTB* and to detect rifampicin resistance.

Xpert MTB/RIF was initially used for high-risk groups, but in 2013 the WHO recommended that the assay be considered the initial diagnostic test for all people requiring investigation for TB ^12^. Between 2010 and 2016, nearly 6,700 Xpert instruments were procured by NTPs in 130 of 145 countries eligible for concessional pricing, and, in 2016 alone, this included 6.9 million test cartridges ^5^. These impressive statistics, however, belie major challenges with the use of Xpert MTB/RIF, such as the need for stable and regular electricity, adequate maintenance, and uninterrupted cartridge supplies ^13^. In many countries, installed instruments are underutilized; the island of Kiribati, which has a significant TB burden, is an illustration of this point ^14^.

NTPs should consider four practical steps to improve systems for TB diagnosis. First, with many programs in resource-poor areas still in the process of scaling up Xpert technology, a mixture of smear microscopy and Xpert MTB/RIF is used. NTPs need to work out how and where these two diagnostic systems are deployed and the most practical and effective algorithms for their use.

Second, NTPs need to have accurate and timely records of numbers presenting with presumptive TB, numbers who submit sputum or other bodily specimens for laboratory investigation, and numbers diagnosed with bacteriologically confirmed TB. This cascade should be routinely monitored and is a fertile area for operational research. A recent review in primary care clinics in South Africa showed that 20% of patients with classic TB symptoms were never screened by health-care staff and that 80% of patients who were screened and identified with TB symptoms failed to submit the necessary sputum specimens ^15^. These system-related deficiencies suggest that much more can be done within the health-care setting to identify patients with presumptive TB and ensure that they are appropriately investigated.

Third, depending on the number of modules per Xpert MTB/RIF instrument (the usual instrument has four modules), it is simple to calculate the number of expected assays that should be performed per instrument per month and then to monitor the number of assays actually performed against this target. Such information is critical for evaluating the proper deployment and effective use of these instruments.

Finally, NTPs must keep abreast of new developments in molecular technology. Xpert MTB/RIF has its limitations, which include suboptimal sensitivity and a high rate of false positivity in low-prevalence settings. To overcome this, the assay was re-engineered to increase diagnostic sensitivity and improve specificity in the detection of rifampicin resistance. The resulting Xpert MTB/RIF Ultra assay is run on the same instrument as Xpert and requires only a software upgrade ^16^. In 2017, based on a technical expert consultation, the WHO recommended the use of Xpert MTB/RIF Ultra in all settings as a replacement for Xpert MTB/RIF ^17^. However, such replacement comes with advantages and disadvantages. A recent multi-center study showed increased sensitivity of Xpert MTB/RIF Ultra, especially in patients with paucibacillary disease and HIV co-infection, but this was at the expense of decreased specificity, especially in those with a history of TB ^18^. Additional work and research are needed to provide solutions to these problems.

Xpert Omni (a portable, battery-operated, single-cartridge, point-of-care device) is currently undergoing field evaluation, and launch is expected in 2019 ^19^. If costs permit (and worryingly these do appear to be rising), this might allow further decentralization of molecular diagnostic technology and improved patient access.

## Provide rapid, complete, and effective treatment to all those diagnosed with tuberculosis

All patients diagnosed with TB should receive prompt, complete, and effective treatment with appropriate nutritional and psycho-social support. In practice, this does not happen. In Africa, Asia, and the Western Pacific, between 4% and 38% of patients with laboratory-detected sputum smear-positive or culture-positive TB fail to start treatment ^20^. This outcome, known as pre-treatment loss to follow-up, appears to be no better with the use of Xpert MTB/RIF, and pre-treatment loss to follow-up varies from 21% in Cameroon to 30% in Myanmar and to 45% in South Africa ^21– 23^. For patients who do get treated, the turn-around time between confirmed diagnosis and treatment initiation can also be lengthy, compromising individual care and increasing the risk of *MTB* transmission within families and communities.

This disconnection between laboratories, treatment facilities, and patients has to change. District TB officers must be held accountable for linking diagnosed patients to treatment. One simple method is to regularly identify all bacteriologically confirmed TB patients in the laboratory register and check that they have been entered into the patient TB treatment register. In the 1990s, the Malawi NTP adopted this system and set a national performance target that at least 90% of diagnosed patients should start standardized treatment ^24^. Time between diagnosis and start of treatment should also be monitored, and targets should be set for what constitutes an unacceptable time interval (for example, more than 7 days). Serious consideration should also be paid to report treatment outcomes on all diagnosed TB patients and not just on those registered for treatment, as is the current procedure. A study in Ghana showed that although treatment success of registered patients with TB was high at 87%, this reduced to 54% when diagnosed patients served as the cohort denominator ^25^. Unless this change is made, NTPs will be unable under routine program conditions to measure the third pillar of the Global TB Plan, namely whether 90% of their diagnosed patients have successfully completed treatment ^9^.

WHO guidelines (2009 and 2017) specify that new patients with drug-susceptible TB be treated with a standardized regimen of isoniazid and rifampicin for 6 months, supplemented with pyrazinamide and ethambutol for the first 2 months ^26, 27^. Standardization of medication dosages by body weight, use of fixed-dose combinations and patient treatment kits, and insistence that drugs be given under direct observation (directly observed therapy, or DOT) have facilitated treatment administration and improved patient adherence to medication. Community- or home-based DOT is recommended, and DOT by trained community volunteers/workers or formal health-care workers is preferred over DOT that is administered by family members. This system, which is followed by NTPs worldwide, has contributed to high treatment success rates of registered patients, and the latest outcome data show a global treatment success of 83% ^5^.

However, there is room for improvement. Treatment outcome definitions were revised by the WHO in 2013 ( Table 3) ^28^. “Not evaluated” is an outcome given to a patient for whom no treatment outcome is assigned, and rates of “not evaluated” can be high. In the 2013 WHO African region cohort, 9% of patients with TB were classified as “not evaluated”, impacting negatively on the overall treatment success rate ^29^. “Not evaluated” includes patients who are transferred out to another treatment unit and whose treatment outcome is unknown. Transfers during anti-TB treatment are common and often are associated with no treatment outcome in the TB treatment register ^30, 31^. District TB officers, using mobile phones and other modern communication methods, can significantly reduce this correctable adverse outcome and ensure that all of their transferred-out patients have a documented outcome in the register.

**Table 3.** Definitions of treatment outcomes for patients with drug-susceptible tuberculosis.

Outcome	Definition
Cure	A bacteriologically confirmed TB patient at the beginning of treatment who was found to be smear- or culture- negative in the last month of treatment and on at least one previous occasion
Treatment completed	A TB patient who completed treatment without evidence of failure but with no record to show that sputum smear or culture results in the last month of treatment and on at least one previous occasion were negative, either because the tests were not done or because the results are unavailable
Treatment failure	A TB patient whose sputum smear or culture is positive at month 5 or later during treatment
Died	A TB patient who dies for any reason before starting or during the course of treatment
Lost to follow-up	A TB patient who did not start treatment or whose treatment was interrupted for two consecutive months or more
Not evaluated	A TB patient for whom no treatment outcome is assigned; this includes patients “transferred-out” to another treatment unit as well as patients for whom the treatment outcome is unknown to the reporting unit
Treatment success	The sum of *cured* and *treatment completed*

TB, tuberculosis. Adapted from reference 28.

## Implement and monitor effective infection control practices

Infection control is the basis for preventing exposure to *MTB* among non-infected individuals in health facilities and other congregate settings. Health facility exposure accounts for a significant proportion of total TB infection risk for patients who repeatedly attend health facilities for chronic care in high-TB burden settings ^32^. Health-care staff are similarly at high risk ^33^. In the high-HIV prevalence areas of Southern Africa, devastating and lethal outbreaks of drug-resistant TB have occurred in the open and crowded hospital wards and out-patient facilities ^34, 35^.

NTPs must pay attention to the WHO TB infection control guidelines which focus on early identification, isolation, and treatment of those with presumptive TB, infrastructure modifications (such as enlarged windows, open skylights, and open-air waiting rooms) to ensure appropriate natural ventilation and air flow, use of ultraviolet germicidal irradiation fixtures (if applicable and affordable), better organization to avoid patient congestion, and provision of personal protective measures for health workers ( Table 4) ^36^. For drug-resistant TB, the introduction of the FAST strategy (find cases actively, separate safely, and treat effectively) has been associated with a significant reduction of hospital-based acquisition of MDR-TB in the Russian Federation ^37^, and this approach should be encouraged in other countries. An important monitoring indicator for effective implementation within health facilities is the number and proportion of health-care workers who develop TB each year, and this should be routinely reported by NTPs.

**Table 4.** Infection control guidelines for reducing transmission of *Mycobacterium tuberculosis* in health facilities.

**Facility-level measures** • Redesign the use of available spaces, renovate existing facilities, or construct new facilities • Conduct on-site surveillance of TB in health workers • Discuss TB transmission with health workers, patients, and visitors • Monitor the package of TB infection control measures
**Administrative measures** • Promptly identify people with TB symptoms, separate potentially infectious and confirmed infectious patients from others, institute cough etiquette and respiratory hygiene, and minimize time spent in health-care facilities • Provide prevention and care interventions for health workers, including HIV prevention, antiretroviral therapy, and isoniazid preventive therapy for HIV-positive health workers
**Environmental protection** • Ensure good natural ventilation (open windows and doors, enlarged or additional windows, and open skylights for cross-ventilation) • Use ultraviolet germicidal irradiation fixtures (if applicable and affordable, especially in cold climates when windows and skylights remain shut)
**Personal protection** • Use particulate respirators especially for managing drug-resistant TB

TB, tuberculosis. Adapted from reference 36.

## Diagnose and promptly treat drug-resistant tuberculosis

Both RR/MDR-TB require prolonged treatment (traditionally up to 24 months) with second-line anti-TB drugs, which are less effective and more costly and are associated with more adverse events compared with first-line drugs ( Table 5) ^38, 39^. About 10% of patients with MDR-TB have additional resistance to fluoroquinolones or second-line injectable agents (termed extensively drug-resistant TB, or XDR-TB) or both. XDR-TB is best treated with regimens that include bedaquiline and linezolid, but even so about one-third of treated XDR-TB patients have unfavorable treatment outcomes of death, failure, and loss to follow-up ^40^. These adverse treatment outcomes contribute to a growing and dangerous problem of patients with programmatically incurable TB. Globally, in 2016, 4% of new cases and 19% of previously treated TB cases had MDR-TB, and nearly half of the world’s cases are in China, India, and the Russian Federation ^5^. Some countries in Eastern Europe and Central Asia have alarming levels of disease; a national prevalence survey in the Ukraine found the proportions of MDR-TB among new and previously treated cases to be 24% and 58%, respectively ^41^.

**Table 5.** Second-line drugs recommended for the treatment of multidrug-resistant tuberculosis.

Drug class	Name of drug
Fluoroquinolones	Levofloxacin Moxifloxacin Gatifloxacin
Second-line injectable agents	Amikacin Capreomycin Kanamycin (Streptomycin)
Other core second-line agents	Ethionamide/Prothionamide Cycloserine/Terizidone Linezolid Clofazimine
Add-on agents	Bedaquiline Delamanid p-aminosalicylic acid Imipenem-cilastatin ^a^ Meropenem ^a^ Amoxicillin-clavulanate ^a^ (Thioacetazone) ^b^

^a^Carbapenems and clavulanate are meant to be used together: clavulanate is available only in formulations combined with amoxicillin. ^b^HIV status must be confirmed as negative before thioacetazone is started. Adapted from references 38 and 39.

Countries with known high levels of MDR-TB should test all new and previously treated cases of TB for rifampicin resistance by using Xpert MTB/RIF. Those with lower drug-resistance burdens must examine their resources and decide, for example, whether to prioritize all previously treated TB cases for Xpert MTB/RIF and work out when to use Xpert MTB/RIF on new cases that are diagnosed through smear microscopy.

Many countries still like to confirm their RR-TB Xpert results with conventional culture and drug sensitivity testing (CDST) in national reference laboratories, and this requires the transportation of sputum specimens from peripheral health facilities. Wherever this system has been assessed, it does not function ^42– 45^, and transportation is the major bottleneck. Other obstacles include the formation of committees to approve MDR-TB treatment and a battery of hematological and biochemical tests which are considered necessary in all settings before treatment can be started. Although these procedures may be necessary when new drugs such as bedaquiline and delamanid are introduced in programmatic settings, they can contribute to diagnosed patients either failing to start treatment or experiencing significant delays. NTPs must decide what to do: whether to streamline the preparatory phase for starting MDR-TB treatment and whether to maintain but fix their national reference laboratory CDST systems or alternatively dispense with CDST and focus on scaling up and decentralizing their diagnostic molecular technology.

This is not an easy decision. The WHO has recently recommended a new short-course regimen of 9 to 12 months for MDR-TB provided that patients have not been treated with second-line drugs and/or have had resistance to fluoroquinolones and second-line injectable agents excluded ^46^. This regimen in eligible patients is well tolerated and, according to observational studies, has excellent treatment success rates both in Asia and in Africa ^47, 48^. Definitive and published results from the STREAM 1 randomized controlled trial comparing the 9-month regimen with a 24-month regimen are awaited.

Currently, conventional CDST and molecular line probe assays (LPAs) performed in laboratories are the only ways of obtaining information on second-line drug resistance. LPAs are more rapid than conventional phenotypic drug sensitivity testing (DST) methods, allowing a rapid diagnosis of either MDR-TB or XDR-TB within 3 days either from sputum submission or after obtaining a pure culture of *MTB*. LPAs are therefore recommended by the WHO as the initial laboratory test instead of phenotypic DST for diagnosing MDR-TB or XDR-TB ^49, 50^. Good functioning and well-resourced laboratories plus well-trained, skilled laboratory technicians are necessary for LPAs to perform well, and thus these assays are generally used only in national reference laboratories.

However, a new automated, cartridge-based assay has been developed that accurately detects *MTB* mutations associated with resistance to isoniazid, fluoroquinolones, and aminoglycosides, and this holds promise as a rapid point-of-care test to guide therapeutic decisions for patients with drug-resistant TB ^51^. This will also facilitate the identification of isoniazid mono-resistance, which may be found in 10% or more of patients and which currently needs conventional CDST or laboratory-based LPA for diagnosis. Isoniazid mono-resistance may be associated with worse treatment outcomes ^52^, and the WHO recommends that high levels of isoniazid mono-resistance warrant a change of regimen to rifampicin, ethambutol, pyrazinamide, and levofloxacin ^53^.

## Diagnose and treat HIV infection and diabetes mellitus

### HIV infection

The HIV epidemic has hampered TB control efforts globally, especially in the high-HIV prevalence areas of Southern and Eastern Africa. Case fatality rates are several times higher in patients with HIV-associated TB compared with those who have just TB ^54^. ART, however, results in excellent immunological and virological responses and a reduction in mortality risk of 64% to 95% ^55^. Cotrimoxazole preventive therapy (CPT) augments this response and, when combined with ART, further reduces mortality and morbidity ^56^.

HIV testing is the gateway to such integrated care, but in 2016 only 57% of notified TB cases globally had a documented HIV test; the figure was highest in the Africa region at 82% ^5^. The WHO recommends ART for all HIV-positive TB patients within the first 8 weeks of starting anti-TB treatment but, although this has improved in recent years, was still only 85% in 2016 ^5^. In 2014, 87% of HIV-positive TB cases globally received CPT ^29^.

NTPs must be bold and aim for 100% HIV testing uptake and 100% CPT and ART for those diagnosed HIV-positive. How this is best achieved is up to countries and depends on how well TB and HIV/AIDS services are decentralized along with the needed clinic infrastructure and trained health-care staff. Approaches need to be contextual and innovative, preferably aiming for a “one-stop-shop” service that integrates TB and HIV care ^57^.

### Diabetes mellitus

People with diabetes mellitus (DM) have a three-times-higher risk of TB compared with the general population ^58– 60^. Both type 1 and type 2 DM increase this risk, but as type 2 disease accounts for 90% or more of the global cases of DM, this type of DM dominates the interaction. In 2012, it was estimated that the number of adult TB cases associated with DM was just over 1 million, similar to what was observed at that time for HIV-associated TB ^60^.

DM adversely affects TB treatment outcomes ^61^. The reasons are not completely understood but include the immunosuppressive and biochemical effects of DM itself, drug–drug interactions, adverse events from medications, suboptimal adherence, and reduced bio-availability of the drugs. The risk of death during TB treatment is almost doubled among those with DM, and this risk increases to about five times when adjustments are made for age and other potential confounders ^61^. Cardiovascular disease could explain an increased rate of deaths within months after starting anti-TB treatment ^62^ and the much higher death rates among DM patients who smoke ^63^. The risks of relapse TB in those who have completed anti-TB treatment are also higher among those with DM compared with those without ^61^, and this further adds to the disease burden. DM is also an independent risk factor for the development of MDR-TB ^64^.

Globally, the prevalence of DM among patients with TB ranges from 1.9% to 45%, and risk factors are older age, urban residence, sedentary life-style, and having a family history of DM ^65^. The WHO and the International Union Against Tuberculosis and Lung Disease now recommend that all patients with TB be systematically screened for DM ^66^, and implementation research within the general health services in India and China has shown the feasibility and effectiveness of screening for DM at the time of registering TB patients for treatment ^67, 68^. There is some evidence that improved management of DM and the use of metformin as the oral anti-diabetes medication reduce the risk of death during TB treatment, although these encouraging findings need confirmation ^69, 70^. With the global pandemic of DM escalating and seemingly out of control ^71^, NTPs need to keep up to date with advances in the field of DM-associated TB and seriously consider setting up routine screening and care for DM.

### Design and implement active case finding strategies for high-risk and vulnerable groups

In most TB endemic settings, TB case detection is based on passive case finding, namely waiting for symptomatic patients to seek health-care. This strategy, as shown consistently in TB prevalence surveys, is inadequate to detect the substantial burden of undiagnosed TB in the community ^72^. Active case finding (ACF) is an additional approach, which proactively seeks to identify patients with TB.

Mathematical models suggest that ACF could increase TB case finding, and the WHO has provided guidelines for the systematic and active screening of high-risk groups ( Table 6) ^73, 74^. In one review, the yields of newly diagnosed TB through ACF were 0.7% in population-based community surveys, 2.2% in contact-tracing studies, 2.3% in mines, 2.5% in prisons, 8.2% in medical and ART clinics, and 8.5% in HIV voluntary and counselling services ^75^. Prisons and health-care facilities in low-resource settings are high-risk areas for intense TB transmission ^76, 77^, and ACF combined with other TB control measures would help to considerably reduce TB incidence in these settings. Interest has recently developed about screening people with DM for TB. There are no global data, but two large studies from China and India within the routine health services found that the yields of TB were significantly higher than those found from passive case finding amongst the general population ^78, 79^.

**Table 6.** World Health Organization recommendations on risk groups for screening.

Strong recommendations ^a^
Household and other close contacts should be systematically screened for active TB
People living with HIV should be screened for TB at each visit to a health facility
Current and past workers in workplaces with silica should be screened for active TB
**Conditional recommendations** ^b^
Systematic screening for active TB should be considered in prisons
Systematic screening for active TB should be considered in those with fibrotic chest radiograph lesions
Systematic screening for active TB should be considered for those seeking or in health-care
Systematic screening for active TB should be considered in other high-risk groups; these include people living in urban slums, homeless people, people living in remote areas, some indigenous populations, and migrants/refugees

TB, tuberculosis. ^a^A strong recommendation is one in which the desirable effects clearly outweigh the undesirable effects and for which screening is judged to be feasible, acceptable, and affordable in all settings. ^b^A conditional recommendation is one in which the desirable effects probably outweigh the undesirable effects. Adapted from reference 74.

To reduce the “missing 4 million TB patients”, NTPs will have to take up ACF, but the scale and focus of what can be done must be contextualized and will be limited by available resources. People living with HIV (PLHIV) and household contacts are easily identifiable high-risk targets and they should be prioritized.

Amongst PLHIV, systematic TB screening (termed intensified case finding) is now an accepted part of the package of care. In 2016, 88,200 (7%) of the 1.3 million PLHIV newly enrolled in HIV care were diagnosed with TB ^5^, although this figure probably underestimates the true burden of TB in this population. Disseminated TB which is more prevalent in those with advanced immunodeficiency is difficult to diagnose in most health-care facilities in resource-poor settings, and autopsy studies show that much of it is diagnosed post-mortem and not recognized or identified during life ^80^. For sick, immunocompromised patients, there is a promising bedside test involving measurement of urine lipoarabinomannan (LAM), one of the cell wall lipopolysaccharide components of *MTB.* The urine test can be carried out with a Determine TB-LAM test strip (Alere, Waltham, MA, USA) with results in 30 minutes. Specificity is high and sensitivity increases as CD4 cell counts decrease to below 100 cells/µL ^81^. Bedside urine LAM (with a positive result guiding the start of anti-TB treatment) on HIV-infected adults admitted to hospital in several African countries resulted in a decrease in mortality at 2 months ^82^. Xpert MTB/RIF can also be used on urine specimens to identify HIV-infected hospitalized patients with TB ^83^. A new screening approach in Malawi and South Africa showed that testing people living with HIV and admitted to hospital with urine LAM and urine Xpert MTB/RIF in addition to sputum Xpert MTB/RIF resulted in increased overall TB diagnosis and treatment and reduced mortality in key subgroups ^84^. Sick HIV-infected patients with pulmonary and disseminated TB often cannot cough up sputum, and the use of urine as a suitable and easy-to-collect specimen for investigation is a novel step forward.

For years, NTPs have tried, with limited success, to screen household contacts of index smear-positive TB patients, focusing on children younger than 5 years of age. A recent systematic review showed that all household contacts (including adults) are at high risk of developing TB ^85^. A cluster randomized trial in Vietnam, with repeated visits to households and assessments by symptoms, physical examination, and chest radiography, has shown the undoubted benefit and high yield of this ACF approach ^86^. NTPs must seriously consider implementing and monitoring this ACF activity.

## Treat latent tuberculosis infection in high-risk groups

ACF aims to identify amongst high-risk groups those with undiagnosed active TB and at the same time this approach can be used to identify and treat those with latent TB infection (LTBI) to prevent them from developing active disease. Thus, ACF is linked to the treatment of LTBI, and the two high-priority groups for the intervention are the same: PLHIV and household contacts of patients with TB. LTBI is diagnosed by the tuberculin skin test or interferon-gamma release assay ^87^. Both tests are challenging to use in programmatic settings and this has often proven to be a bottleneck to treating LTBI ^88^. Updated guidance from the WHO on the programmatic management of LTBI may help to move forward this important but generally neglected aspect of TB control ( Table 7) ^89^.

**Table 7.** Guidance for diagnosing and treating latent tuberculosis infection.

At-risk populations for LTBI testing and treating	Adults, adolescents, children, and infants living with HIV HIV-negative household contacts of persons with bacteriologically confirmed TB (children, adolescents, and adults) Other HIV-negative at-risk groups, according to resources
Testing for LTBI	Tuberculin skin test (TST) or interferon-gamma release assay (IGRA) wherever possible People living with HIV with a positive test benefit more from preventive treatment: where testing is possible, this can be used to identify such individuals TST or IGRA is not a requirement for initiating preventive treatment in people living with HIV or child household contacts younger than 5 years of age
Treatment options for LTBI	Isoniazid monotherapy daily for 6 months Rifampicin and isoniazid daily for 3 months as alternative to isoniazid Rifapentine and isoniazid weekly for 3 months as alternative to isoniazid Isoniazid monotherapy daily for at least 36 months in people living with HIV in high-TB burden settings, regardless of whether they are receiving antiretroviral therapy
Preventive therapy for contacts of patients with multidrug-resistant TB	Treatment options based on individual risk assessment; strongly consider use of levofloxacin or moxifloxacin

LTBI, latent tuberculosis infection; TB, tuberculosis. Adapted from reference 89.

PLHIV deserve special mention. ART reverses the immune dysfunction associated with HIV and, as a result, has a potent TB-preventive effect ^90^. At the program level, the increasing ART coverage in people living with HIV in Swaziland, Zimbabwe, and Malawi has been associated with a decrease in national TB case notification rates for both HIV-positive and HIV-negative persons ^91– 93^. Randomized controlled trials have shown that the effects of ART in reducing TB incidence may be augmented with the addition of isoniazid preventive therapy (IPT) ^94, 95^. There is further evidence to suggest that in high-TB exposure environments IPT might have to be given indefinitely for sustained benefit: in this context, it is likely that IPT prevents new infection or reinfection from developing into active disease ^96^. The number of PLHIV who received IPT in 2016 was 940,269; the largest proportion (41%) came from South Africa ^5^. Despite progress in some other countries (for example, Malawi, Zimbabwe, and Mozambique), much remains to be done. NTPs need to liaise closely with National HIV/AIDS programs in this regard and decide whether and how best to scale up IPT alongside ART.

## Engage with the private-for-profit sector

Many resource-poor countries have large and expanding private-for-profit health sectors, and many people with TB seek and receive care from private providers. This is especially so in Asia, but it is also a growing challenge in other parts of the world, including sub-Saharan Africa ^97, 98^. In recent years, the WHO has begun to address the issue of private-for-profit providers in TB prevention and care through an evolving global strategy called Public-Private Mix (PPM) ^98^. Central to the concept of PPM and the delivery of quality TB care is the mix of “clinical tasks” (referrals of symptomatic patients, diagnosis, and prescribing treatment) and “public health tasks” (quality assurance, patient follow-up and support, recording, and reporting) and the linkage of private sector providers to local public sector TB programs. Since 2009, high-TB burden countries have reported on the contribution of PPM to TB case notifications, and in 2016 this was approximately 17% in India, 26% in Pakistan, 28% in Bangladesh, and 16% in Myanmar ^5^. These reports, however, do not reflect ground realities where PPM is often limited to urban areas and a small proportion of targeted providers ^98^. PPM must become an important partner of NTPs; the persisting challenges and potential ways of improving the service are outlined in Table 8. New guidance provided by the WHO in 2017 should help in this regard ^99^.

**Table 8.** Challenges and potential solutions for scaling up private-public mix.

Challenges
Overburdened TB Programs
Fragmented private sector with limited capacity to undertake non-clinical tasks
Weak capacity of public sector to oversee and manage contracts with private organizations
Agreed regulations (e.g. mandatory TB case notifications) are not enforced
Insufficient financing
Potential solutions
Get governments to commit to invest in the private sector
Consider the financing of private-public mix through universal health coverage
Set up permanent working linkages between TB programs and private health facilities
Use intermediary organizations to design, manage, and supervise contracts
Engage in business-friendly approaches using smart application of new digital technologies
Enforce regulations (mandatory TB case notifications through web-based systems: restrictions on private over-the-counter sales of anti-TB drugs)

TB, tuberculosis. Adapted from references 97 and 98.

## Empower and strengthen the central unit of the National TB Programme

NTPs need a strong central unit to provide leadership, planning, training, drug and consumables forecasting, procurement, budgeting, supervision, monitoring and evaluation, and operational research. Ways to integrate TB program activities with other disease-related areas such as HIV/AIDS and DM need to be developed and implemented. NTPs also need to engage regularly with other stakeholders and government departments to make a strong case for reducing poverty and overcrowding and tackling the other key social determinants of TB.

Three areas need emphasis: supervision, research, and finances. Regular, supportive, and structured data-driven supervision coordinated by the central unit is essential to TB control. Supervision within communicable disease control programs helps to check whether guidelines are implemented, drug stocks are sound, records are accurate, and cohort analysis at peripheral levels is correct and meaningful ^7, 100, 101^. In all of this, supervisors must recognize the potentially perverse nature of targets and ensure that NTPs do not drown in fake numbers ^102^.

Operational research needs to be firmly embedded within the NTP, as this enables programs to carry out local research that contributes to knowledge, tools, interventions, and strategies that can enhance the quality and coverage of service delivery and help inform better policy and practice ^103, 104^. The placement of operational research fellows within the central unit of NTPs greatly facilitates the implementation and value of this work ^105, 106^. NTPs must also form partnerships with academia to facilitate the conduct of meaningful clinical or cluster randomized trials, as demonstrated by the recent household contact assessment in Vietnam ^86^.

In 2017, most of the funding for TB control efforts was provided by domestic sources (84% of the global total of USD $6.9 billion), although in low-income countries international donor funding, mainly through the Global Fund to fight AIDS, Tuberculosis and Malaria, exceeded domestic funding and this is likely to remain the case ^5^. Development assistance for health is unlikely to increase in the future, so NTPs need to advocate strongly for domestic funding, ensure that spending is efficient and not wasteful of resources, and keep abreast of new innovative international financing instruments which can benefit global health ^107^. NTPs in low- and middle-income countries need to emphasize that the diagnosis and treatment of drug-sensitive and drug-resistant TB are very cost-effective when compared with the economic cost and productivity losses that may result from the disease ^108^.

## Conclusions

NTPs on their own cannot end the global TB epidemic, which will require accelerated socio-economic development, poverty alleviation, social protection, actions on other determinants of TB, strengthening of health systems, and expansion of universal health coverage. For this multi-sectoral response, there will need to be close engagement of communities, civil society organizations, the private sector, and different government ministries. New tools for preventing, diagnosing, and treating TB are also urgently needed ^6^.

Nevertheless, NTPs play a crucial role. This article has highlighted the basic TB control principles that need to be adhered to, not just in planning but also in implementation. The glaring gaps in the delivery of diagnostic, treatment, and preventive services along with our remedial recommendations are summarized in Table 9. The quality of delivered care often falls short of international standards in both public and private sectors, and NTPs must step up to the plate, systematically measure and improve quality of care, and invest in quality improvement programs ^109^.

**Table 9.** Challenges and recommendations for National TB Programmes.

Category	Challenges	Proposed remedies
End TB Milestones/Targets on TB incidence and mortality	Presented globally as percentage reductions compared with 2015	Change percentage reductions into absolute “national” numbers against which to measure progress and present them for all levels of the health system
Optimize prompt and accurate diagnosis of TB	Inefficient use of smear microscopy and Xpert MTB/RIF	Develop algorithms for best use of smear microscopy/Xpert MTB/RIF; monitor numbers being screened, tests done, and results; monitor operational capacity of MTB/RIF instruments; keep abreast of new diagnostic molecular technology
Provide rapid, complete, and effective treatment to all those diagnosed with TB	Poor linkage of diagnosis and treatment; wrong denominator for measuring treatment outcomes; high rates of “not evaluated”	Monitor and improve linkage to care; use diagnosed TB patients as the denominator for treatment outcomes; obtain treatment outcomes for transfer-out patients
Implement and monitor effective infection control practices for *MTB*	Poor infection control practices and monitoring of TB infection control	Implement and monitor World Health Organization-recommended guidelines; monitor rates of TB in health workers
Diagnose and treat drug-resistant TB	Poor linkage to care; continued use of inefficient CDST systems at national laboratories; long duration of therapy	Monitor and improve linkage to care; prioritize and plan for more efficient use of Xpert MTB/RIF; aim for short 9- to 12-month multidrug-resistant TB treatment; keep abreast of new diagnostic molecular technology
Diagnose and treat HIV infection and diabetes mellitus	HIV and diabetes increase TB mortality; suboptimal uptake of HIV testing, ART, and CPT; limited screening of TB patients for diabetes	Aim for 100% uptake of HIV interventions; scale up screening of TB patients for diabetes and provide optimal diabetes care during anti-TB treatment
ACF and treatment of LTBI	Poor programmatic implementation of ACF and treatment of LTBI	Design and implement strategies with particular focus on people living with HIV and household contacts
Engage with private-for-profit sector	Poor implementation of the PPM strategy	Focus on ways to improve the PPM strategy, especially in Asia
Empower and strengthen central units of TB Programs	Weak central units	More focus on data-driven supportive supervision, operational research, and sustained financing

ACF, active case finding; ART, antiretroviral therapy; CDST, culture and drug susceptibility testing; CPT, cotrimoxazole preventive therapy; LTBI, latent tuberculosis infection; MTB, *Mycobacterium tuberculosis*; PPM, public-private mix; TB, tuberculosis.

New paradigms also have to be embraced or else the planned new trajectories for reducing TB incidence and mortality will never be achieved. As new tools are developed, there needs to be a fast-track system for getting them pilot-tested and scaled up in the field, and this will require a broad-minded approach from national TB leadership. The clock is ticking and we need to think and act differently!

## Disclaimer

The views expressed in this document are those of the authors and may not necessarily reflect those of their affiliated institutions.
